# Timing of Pharyngeal Swallow Events in Chagas’ Disease

**DOI:** 10.14740/gr616w

**Published:** 2014-07-31

**Authors:** Carla Manfredi dos Santos, Rachel de Aguiar Cassiani, Weslania Viviane do Nascimento, Roberto Oliveira Dantas

**Affiliations:** a Department of Medicine of the Medical School of Ribeirao Preto, University of Sao Paulo, Ribeirao Preto, SP, Brazil

**Keywords:** Chagas’ disease, Swallowing, Deglutition, Swallowing control, Megaesophagus, Pharynx

## Abstract

**Background:**

Esophageal involvement by Chagas’ disease causes a significative decrease in the number of neurons of the esophageal myenteric plexus, causing an impairment of esophageal motility with the same alterations described in primary esophageal achalasia. There is also a longer duration of pharyngeal transit, which might be consequent of the involvement of the central control of swallowing by the disease, or an adaptation of the pharynx to the difficult bolus transit through the esophagus, which could contribute to the complaint of dysphagia.

**Methods:**

We studied, by videofluoroscopy, the sequence and timings of pharyngeal bolus transit in 16 patients with esophageal involvement by Chagas’ disease and 12 healthy volunteers. Each subject swallowed in duplicate 5 mL and 10 mL of liquid and paste boluses.

**Results:**

There was no difference between Chagas’ disease patients and normal volunteers in the sequence and timing of events associated with pharyngeal bolus flow, for liquid and paste boluses, and for 5 mL and 10 mL.

**Conclusion:**

The timing and sequence of swallow pharyngeal events of patients with Chagas’ disease do not differ from that of control subjects, which suggested that the central control of swallowing is not impaired by the disease.

## Introduction

The esophageal involvement by Chagas’ disease is one of the most frequent causes of dysphagia in the South American population [1, 2]. It is consequent of the pathological involvement of the esophageal myenteric plexus, which has a significantly lower number of neurons [3], causing the impairment of esophageal motility, with the same alterations described in primary esophageal achalasia, i.e. partial or complete absence of lower esophageal sphincter opening and aperistalsis in the esophageal body, and megaesophagus [2, 4].

However, the pharyngeal phase of swallowing may also be involved, with longer duration of pharyngeal transit [10]. A previous study suggested that the CNS control of pharyngeal transit is not impaired in these patients [11, 12].

Our aim in this investigation was to evaluate the timing and sequence of pharyngeal swallowing events in patients with esophageal involvement by Chagas’ disease, with the hypothesis that, although possible, there is no alteration of the timing and sequence of these events when compared to healthy volunteers.

## Materials and Methods

We studied 16 patients with Chagas’ disease and 12 healthy volunteers. The group of Chagas’ disease patients consisted of nine women and seven men, aged 30 - 65 years, mean 52.4 years, with dysphagia and a positive serologic examination for Chagas’ disease. Esophageal contrast radiography found esophageal retention of 100% barium sulfate for more than 30 s after ingestion of a volume of 100 mL, with an increase in distal esophageal diameter (higher than 4 cm) in five patients. The control group had 12 asymptomatic healthy volunteers, seven women and five men, aged 33 - 66 years, mean 53.4 years, who had never lived in endemic areas for Chagas’ disease. Subjects with heart disease, diabetes, hypertension, respiratory, neurological or renal diseases or those who were taking drugs were excluded from both groups. No subject included in the investigation had been previously treated for esophageal or gastric diseases, nor had dementia, confusion, sensory or motor deficits.

The study was conducted at the University Hospital of the Medical School of Ribeirao Preto, University of Sao Paulo, and the protocol of the investigation was approved by the Human Research Committee of the University Hospital of Ribeirao Preto. Written informed consent was obtained from each participant.

Videofluoroscopy evaluation of swallowing was done with an Arcomax angiograph unit (Phillips, model BV 300, Veenpluis, The Netherlands). The images were recorded at 60 frames per second using the Ever Focus model EDSR 100 V1.2 digital processing system (Taipei, Taiwan) with a DVR monitor (Ever Focus) and a digital clock that indicates time in minutes, seconds and the number of frames on each video frame. Mouth, pharynx and proximal esophagus were imaged in lateral projection, with the subjects sitting in a chair with both feet on the floor. Boluses of 5 mL and 10 mL of a liquid and 5 mL and 10 mL of a paste were swallowed in duplicate. For the liquid bolus, barium sulfate (Bariogel^®^ 100%, Laboratory Cristalia, Itapira, SP, Brazil) was offered with the aid of a spoon. For the paste bolus, we added 30 mL of 100% liquid barium sulfate to 3 g of the food thickener Nutilis (Nutricia Cuyk B.V., DJ Cuyk, The Netherlands), which was also offered with a spoon. The liquid bolus had a consistency of thick liquid and the paste bolus the nectar consistency.

The following features were timed: 1) onset of tongue base anterior movement (OTB); 2) arrival of the bolus head at the fauces, considered as the onset of the pharyngeal phase of swallowing (OPP); 3) end of oral transit, when the bolus tail arrived at the fauces (EOT); 4) onset (OHM) and end (EHM) of hyoid movement; 5) onset (UESO) and offset (UESC) of upper esophageal sphincter (UES) opening. The swallows were performed in the sequence: two 5 mL liquid, two 10 mL liquid, two 5 mL paste, two 10 mL paste, with an interval of 30 - 60 s between swallows. The reference for all timing measurement was OPP, considered as zero moment.

Statistical analysis was done by using a linear model with mixed effects [13]. The tests were performed at the Center of Quantitative Analysis of the Medical School of Ribeirao Preto, University of Sao Paulo. The results are reported, in milliseconds (ms), as mean and standard deviation (SD). A P-value < 0.05 was considered significant.

## Results

**Figure 1:**
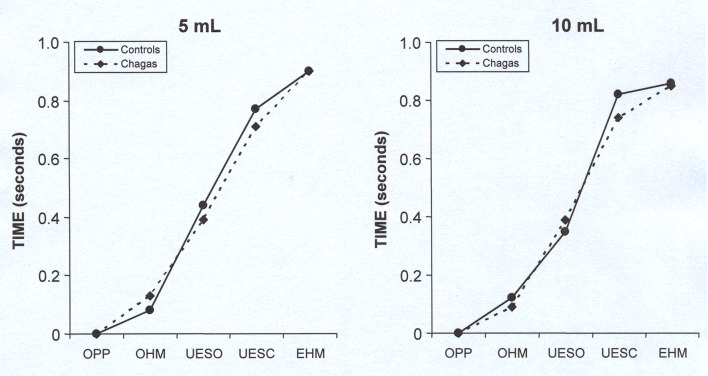
Mean of the timing of pharyngeal swallow events, in seconds, in patients with Chagas’ disease and control subjects after swallowing of 5 mL and 10 mL of liquid bolus. P > 0.05, controls vs. Chagas. OPP: onset of pharyngeal phase; OHM: onset of hyoid movement; UESO: upper esophageal sphincter opening; UESC: upper esophageal sphincter closing; EHM: end of hyoid movement.

**Figure 2:**
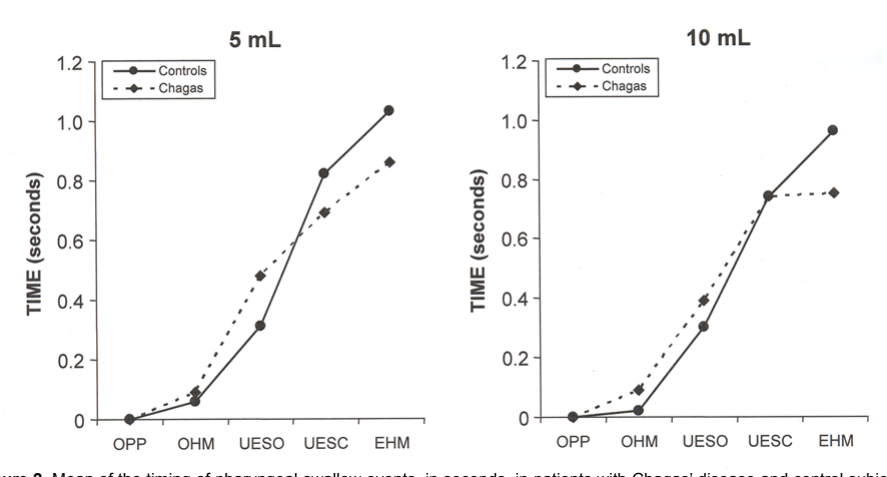
Mean of the timing of pharyngeal swallow events, in seconds, in patients with Chagas’ disease and control subjects after swallowing of 5 mL and 10 mL of paste bolus. P > 0.05, controls vs. Chagas. OPP: onset of pharyngeal phase; OHM: onset of hyoid movement; UESO: upper esophageal sphincter opening; UESC: upper esophageal sphincter closing; EHM: end of hyoid movement.

There was no difference between Chagas’ disease patients and normal volunteers in the sequence and timing of events associated with the pharyngeal bolus flow (P **>** 0.05), for liquid (Table 1) and paste (Table 2) boluses, and for 5 mL and 10 mL. The figures show the sequence and timing, as mean, of pharyngeal events with swallows of the liquid bolus (Fig. 1) and with swallows of the paste bolus (Fig. 2).

**Table 1:** Timing of Swallowing Events, in Seconds, After Swallows of 5 mL and 10 mL Liquid Bolus, in Patients With Chagas’ Disease (n = 16) and Controls (n = 12), Mean (SD)

	5 mL		10 mL	
	Controls	Chagas	Controls	Chagas
OTB	-0.22 (0.14)	-0.27 (0.36)	-0.22 (0.24)	-0.33 (0.29)
OPP	0	0	0	0
EOT	0.25 (0.26)	0.38 (0.31)	0.29 (0.24)	0.37 (0.33)
OHM	0.08 (0.20)	0.13 (0.34)	0.12 (0.33)	0.09 (0.36)
UESO	0.43 (0.31)	0.39 (0.28)	0.35 (0.32)	0.39 (0.34)
UESC	0.76 (0.17)	0.72 (0.26)	0.82 (0.45)	0.74 (0.24)
EHM	0.89 (0.13)	0.90 (0.32)	0.86 (0.42)	0.85 (0.25)

P > 0.05, controls vs. Chagas. OTB: onset of tongue base anterior movement; OPP: onset of pharyngeal phase; OHM: onset of hyoid movement; EOT: end of oral transit; UESO: upper esophageal sphincter opening; UESC: upper esophageal sphincter closing; EHM: end of hyoid movement.

**Table 2:** Timing of Swallowing Events, in Seconds, After Swallows of 5 mL and 10 mL Paste Bolus, in Patients With Chagas’ Disease (n = 16) and Controls (n = 12), Mean (SD)

	5 mL		10 mL	
	Controls	Chagas	Controls	Chagas
OTB	-0.44 (0.29)	-0.37 (0.34)	-0.53 (0.37)	-0.41 (0.36)
OPP	0	0	0	0
EOT	0.29 (0.24)	0.33 (0.28)	0.36 (0.33)	0.53 (0.25)
OHM	0.07 (0.21)	0.09 (0.34)	0.04 (0.34)	0.12 (0.33)
UESO	0.31 (0.21)	0.49 (0.32)	0.30 (0.25)	0.39 (0.32)
UESC	0.82 (0.14)	0.69 (0.34)	0.77 (0.38)	0.78 (0.35)
EHM	1.04 (0.25)	0.86 (0.38)	0.96 (0.52)	0.80 (0.37)

P > 0.05, controls vs. Chagas. OTB: onset of tongue base anterior movement; OPP: onset of pharyngeal phase; OHM: onset of hyoid movement; EOT: end of oral transit; UESO: upper esophageal sphincter opening; UESC: upper esophageal sphincter closing; EHM: end of hyoid movement.

## Discussion

The results did not show difference in the timing and sequence of pharyngeal events between patients with Chagas’ disease and controls. It is a demonstration that, in the chronic phase, the central control of swallowing should not be compromised by Chagas’ disease.

Esophageal diseases may cause pharyngeal and UES function abnormalities [17]. Achalasia of the lower esophageal sphincter may be associated with alterations of the pharynx and UES function [18, 19], mainly an incomplete UES opening and increase in UES residual pressure [20, 21]. There is no demonstration that the opening of the UES is impaired in achalasia secondary to Chagas’ disease. Primary achalasia and achalasia caused by Chagas’ disease have differences in pathophysiology and in esophageal motility impairment [22], but both have as consequence esophageal dilation, dysphagia and regurgitation, with the same surgical and clinical treatment [23].

There is a precise and complex control of swallowing by the CNS [24], which coordinates respiration and deglutition to avoid difficulty in swallowing and respiratory disconfort. During swallows the control of muscle movements is done by cranial and peripheral nerves which are coordinated within the brain stem, mainly medulla oblongata, with sensory nuclei, motor nuclei and interneurons [25]. Impaired sensory or motor ability at any level impairs the efficiency of swallow physiology [25].

In Chagas’ disease, the oral and pharyngeal phases of swallowing seem to be coordinated with and controlled by the CNS [26]. In this situation there is an increase in intra-esophageal pressure [20] which causes a bolus flow resistency. The central control of swallowing makes adaptations to permit a safe swallow and the ingestion of liquid and solid foods. Dysphagia, regurgitation, heartburn and chest pain should be related with esophageal motility disorders. The CNS, which controls the oral-pharyngeal phases of swallowing, seems not to be involved by the disease.

This observation has importance on the treatment of these patients. The focus of treatment of esophageal disease is on the lower esophageal sphincter, with injection of botulin toxin, pneumatic balloon dilation and laparoscopic, open or endoscopic myotomy [23]. The treatment of oral-pharyngeal dysphagia involves postural strategies, changes in bolus volume or viscosity, sensorial enhancement strategies, neuromuscular praxis, specific swallowing maneuvers and surgical or drug-based management of the UES [27]. The results indicated that patients with Chagas’ disease do not need these options of treatment. The long experience in the treatment of the esophageal manifestations of the disease do not need to change and incorporate the options of treatment for oral-pharyngeal dysphagia, at least with the knowledge we have at this moment.

We concluded that the timing and sequence of swallowing pharyngeal events of patients with Chagas’ disease do not differ from that of control subjects, which suggested that the central control of swallowing is not impaired by the disease.
